# ggCyto: next generation open-source visualization software for cytometry

**DOI:** 10.1093/bioinformatics/bty441

**Published:** 2018-06-01

**Authors:** Phu Van, Wenxin Jiang, Raphael Gottardo, Greg Finak

**Affiliations:** Vaccine and Infectious Disease Division, Fred Hutchinson Cancer Research Center, Seattle, WA, USA

## Abstract

**Motivation:**

Open source software for computational cytometry has gained in popularity over the past few years. Efforts such as FlowCAP, the Lyoplate and Euroflow projects have highlighted the importance of efforts to standardize both experimental and computational aspects of cytometry data analysis. The R/BioConductor platform hosts the largest collection of open source cytometry software covering all aspects of data analysis and providing infrastructure to represent and analyze cytometry data with all relevant experimental, gating and cell population annotations enabling fully reproducible data analysis. Data visualization frameworks to support this infrastructure have lagged behind.

**Results:**

*ggCyto* is a new open-source BioConductor software package for cytometry data visualization built on *ggplot2* that enables ggplot-like functionality with the core BioConductor flow cytometry data structures. Amongst its features are the ability to transform data and axes on-the-fly using cytometry-specific transformations, plot faceting by experimental meta-data variables and partial matching of channel, marker and cell populations names to the contents of the BioConductor cytometry data structures. We demonstrate the salient features of the package using publicly available cytometry data with complete reproducible examples in a Supplementary Material.

**Availability and implementation:**

https://bioconductor.org/packages/devel/bioc/html/ggcyto.html

**Supplementary information:**

Supplementary data are available at *Bioinformatics* online.

## 1 Introduction

Cytometry (FCM) is the primary assay for immune monitoring in clinical and research applications (Maecker *et al.*, 2012). Pipelines must handle preprocessing, quality control, analysis (i.e. cell clustering or manual partitioning into homogeneous groups) (O(oups)e*et al.*, 2013; Saeys *et al.*, 2016) and visualization. Proprietary platforms, including FlowJo (Ashland, OR), WinList, FCSExpress and DIVA are the de-facto standards for end-to-end FCM data analysis. Other programming frameworks like Matlab (Matlab 7.0.4, Natick, MA: MathWorks) and Mathematica (Mathematica 9.0, Champaign, IL: Wolfram Research) provide functionality for data import and exploration [indeed, SPADE (Qiu *et al.*, 2011) was initially developed for MATLAB], but lack the general abstraction of cytometry-specific data structures helpful for data analysis. Open-source projects like R/BioConductor (R/BioC) (Gentleman *et al.*, 2004; Ihaka and Gentleman, 1996) and Python provide FCM functionality through user-contributed packages (Frelinger *et al.*, 2012). Currently 47 open source software packages in BioConductor are tagged for ‘FlowCytometry’ (http://bioconductor.org/packages/release/BiocViews.html) but only *flowViz* (Sarkar *et al.*, 2008) is visualization-centric and doesnli support the core BioConductor data structures used to store analyzed, gated and annotated, single-cell FCM data (see Supplementary Material). Other packages focus on different aspects of automated analysis.

We introduce *ggcyto*, a BioConductor package for building reproducible FCM visualizations programmatically. It is built on *ggplot2* (Wickham, 2009) and supports the core BioConductor cytometry data structures making it compatible with any package using those structures (see Supplementary Material).

## 2 ggCyto

### 2.1 Basic principles

**Fig. 1.**
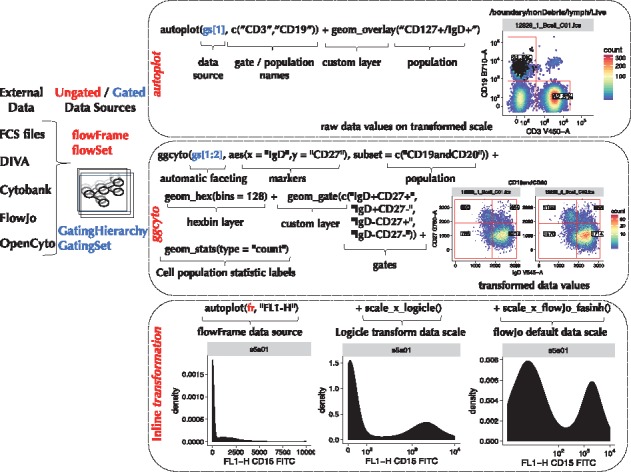
*ggcyto* is compatible with ungated and gated data sources represented by the core BioConductor FCM data structures (flowSet/flowFrame and GatingSet/GatingHierarchy). Plots can be constructed using the (1) *autoplot* or (2) *ggcyto* APIs, giving users more control. Custom layers control cytometry-specific plot elements including 3) data transformation

To construct a plot with *ggcyto* users specify a *data source* (Fig. 1), and, analogous to *ggplot2*, they map plot elements to variables in the *data source.* With *ggcyto* however, users map plot axes to *flow parameters* (e.g. channels or markers), specify the *cell population* to plot, specify cytometry-specific axis transformations and potentially specify *gates* (e.g. elements defining cell populations) to add to the plot. These elements are built up via *layers* and are referred by *name*, mapping directly to quantities (i.e. data) in the *data source.* For ease of use, *ggcyto* supports partial string matches (Fig. 1 and Supplementary Material), particularly useful for identifying complex channel names or cell populations.

### 2.2 Availability

*ggcyto* is open-source and available on GitHub and BioConductor (https://github.com/RGLab/ggcyto/releases/tag/v1.9.5 and https://bioconductor.org/packages/devel/bioc/html/ggcyto.html).

### 2.3 Quick plotting with the *autoplot* API

The *autoplot* API is a quick way to build plots. It makes most of the plot decisions for the user based on domain knowledge and information encoded in the *data source* (Fig. 1 and Supplementary Material). For example, passing a *GatingHierarchy* and a vector of cell population names (defined by gated cell populations in the *GatingHierarchy*) creates a faceted array (one panel for each sample) of two-dimensional density plots (using hexagonal binning) of the *parent cell population* projected onto the dimensions of any *gates* defining those cell subsets (Fig. 1, Supplementary Material). The ‘CD3’ and ‘CD19’ populations shown in Figure 1 are named cell populations defined by gates in the *GatingHierarchy.* They should not to be confused with markers of the same name. Two-dimensional densities are chosen by *autoplot* because the gates defining the CD3 and CD19 cell populations are two dimensional. In cases where gates defining a cell population are one dimensional, a one-dimensional density would be plotted. In this sense, *autoplot* is context aware, selecting *geom*s appropriate for visualizing the desired cell population.

Analogously, *autoplot* can be used to create plots from *flowSet* and *flowFrame* objects (for ungated data) or *GatingHierarchy* and *GatingSet* objects (for gated data, Fig. 1 and Supplementary Material). In the case of ungated data, the user specifies the channels/markers to visualize, rather than the cell population (since the latter is not defined).

### 2.4 Customizing plots with cytometry-specific layers

The *ggcyto()* API provides greater flexibility and customization than *autoplot* (Fig. 1). When using *ggcyto*, the *layers* and defaults selected by *autoplot* are decisions left to the user. Leveraging *ggcytog*s cytometry-specific *layers* and *geoms*, the user builds the plot (Fig. 1 and Supplementary Material) to include the gates, *overlays* (e.g. backgating), data or axis transformations, cell subpopulations and cell subpopulation statistics of interest, and specifies the faceting of plots by metadata annotations (see Supplementary Material). The *ggcyto* API can be particularly useful to project cell populations onto other markers (i.e. not necessarily those on which the populations are defined). The support for data transformations in *ggcyto* is 2-fold: *ggcyto* can transform the underlying data (Fig. 1), or it can transform the axes using the transformation stored in the *data source* (Fig. 1). These approaches are demonstrated in the Supplementary Material.

## 3 Examples

The functionality of *ggcyto* is demonstrated using the Lyoplate dataset from FlowCAP 4 (Finak *et al.*, 2016) available in the *flowWorkspaceData* R/BioConductor package and on the ImmuneSpace portal (Brusic *et al.*, 2014) (see the Supplementary Material for link to this data on ImmuneSpace), as well as the graft versus host disease (GvHD) data available in the *flowCore* R/BioConductor package. Reproducible examples with R code are in the Supplementary Material and available at http://rglab.org/ggcyto/. In future, additional cytometry data may be available via the more modern AnnotationHub or ExperimentHub resources (Morgan *et al.*, 2016; Pasolli *et al.*, 2017).

## 4 Conclusion

The *ggcyto* package provides a powerful and unified visualization interface to complex, ungated or gated, annotated cytometry data structures and provides a key component of a reproducible research workflow. Specifically, the package allows for easy visualization of specific cytometry cell populations and gates, on the fly data and axis transformation, back-gating visualization and easy faceting by study metadata in order to explore variability in an experiment. User-friendliness is made possible through fuzzy name matching, lazy data loading and context-sensitive behavior that aims to capture ‘what the user means to do’ most frequently. Areas for future developments are highlighted in the Supplementary Material.

## Supplementary Material

Supplementary Information
