# Supplementary material for: ggCyto: next generation open-source visualization software for cytometry
Source: Bioinformatics. 2018 Jun 1;34(22):3951–3. doi: 10.1093/bioinformatics/bty441 (PMC6223365; doi:10.1093/bioinformatics/bty441)
Supplement: Supplementary Information [file bty441_supplementary_information.pdf]

Supplementary Information for ‘ggcyto: Next generation visualization software for flow cytometry’

Phu T. Van, Mike Jiang, Raphael Gottardo & Greg Finak

May 21, 2018

Contents

1 Background
2 Overview
2.1 BioConductor flow cytometry tools
2.2 ggcyto supported data structures.
2.3 Support for external packages.
2.4 Interaction with ggplot and the core BioConductor FCM data structures.
2.5 Principles of ggcyto.
2.6 Lazy loading of data
2.7 Additional strategies for plotting large data
2.8 Data sets
3 Reproducing figures from the original manuscript.
3.1 Reproducing the plots from Figure 1.
3.1.1 The `autoplot` API
3.1.2 The `ggcyto` API.
3.1.3 Transforming the data.
4 Additional ggcyto features.
4.1 Context-aware plots.
4.2 Changing `geom` s.
4.3 Using gated data.
4.4 Visualizing back-gating.
4.5 Transforming the axes.
4.6 Using the `ggcyto` interface.
4.7 Faceting plots by experimental metadata.
4.8 Substituting data with the `%+%` method.
4.9 Faceting all markers.
4.10 Future Work
References

```r
knitr::opts_chunk$set(echo = TRUE)
#load the ggcyto package
library(ggcyto)
```

```
## Loading required package: ggplot2
```

```
## Loading required package: flowCore
```

```
## Loading required package: ncdfFlow
```

```
## Loading required package: RcppArmadillo
```

```
## Loading required package: BH
```

```
## Loading required package: flowWorkspace
```

```r
#load flowWorkspaceData
library(flowWorkspaceData)
#load flowWorkspace for importing FlowJo workspaces
library(flowWorkspace)
#for tables
library(xtable)
#for combining plots
library(cowplot)
```

```
##
## Attaching package: 'cowplot'
```

```
## The following object is masked from 'package:ggplot2':
##
##     ggsave
```

1 Background

Open source software for computational cytometry has gained in popularity over the past few years. Efforts such as FlowCAP, the Lyoplate and Euroflow projects have highlighted the importance of efforts to standardize both experimental and computational aspects of cytometry data analysis. The R/BioConductor platform hosts the largest collection of open source cytometry software covering all aspects of data analysis. It provides complex data structures and uses state of the art technologies to store cytometry data with all the relevant experimental, gating, and cell population annotations. While the number of algorithms available in Bioconductor for cytometry data analysis has significantly increased over the past decade, the cytometry specific visualization frameworks available in Bioconductor for these types of data have lagged behind.

2 Overview

This Supplementary Information document for the paper "ggcyto: Next generation visualization software for flow cytometry" shows how to use the BioConductor package `ggcyto` to visualize flow cytometry data and reproduces the plots from the original publication, together with code.

2.1 BioConductor flow cytometry tools

The BioConductor project has, at the time of writing, 47 different packages tagged as "Flow Cytometry" related.

2.2 ggcyto supported data structures.

`ggcyto` supports the core flow cytometry data structures in R/Bioconductor: `flowFrame` and `flowSet` (defined in the `flowCore` package) store ungated data and are created when an FCS or set of FCS files are read into R/BioConductor. The `GatingSet` and `GatingHierarchy` (defined in the `flowWorkspace` package) store gated data. They are created when FCS files together with a **workspace** defining the gating (e.g. from FloJo, DIVA, or CytoBank) are imported together. The `GatingHierarchy` stores a single sample, while the `GatingSet` stores a collection of samples.

Of the 47 cytometry tagged packages on BioConductor, two (including `ggcyto`), are focused on visualization. `flowViz`, the original BioConductor cytometry visualization software has not been updated to support the new cytometry data structures that hold gated and analyzed data. These data structures are part of thre three core cytometry infrastructure packages: flowWorkspace (https://doi.org/doi:10.18129/B9.bioc.flowWorkspace), flowCore (https://doi.org/doi:10.18129/B9.bioc.flowCore) (Finak et al. (2014)), (Hahne et al. (2009)), and ncdfFlow (https://doi.org/doi:10.18129/B9.bioc.ncdfFlow) (M. Jiang et al. (2011)).

2.3 Support for external packages.

`ggcyto` borrows ideas from `flowViz` but greatly enhances functionality and usability through the **grammar of graphics** (Wickham (2009)) by building on **ggplot** to allow direct interaction with the core BioConductor flow cytometry data structures. Its use of established core cytometry data structures in BioConductor enables ggcyto to interact with any R package that uses them.

2.4 Interaction with ggplot and the core BioConductor FCM data structures.

To facilitate the use of core data structures with ggcyto, the package wraps the **ggplot** S3 class in an abstract S4 class that provides support for both ungated (`flowFrame` or `flowSet`) or gated (`GatingSet` or `GatingHierarchy`) data sources and overloads ggplot's **fortify()** method to generate plots from these four core infrastructure objects. In ggcyto, the default "`ggplot::+`" operator is also overloaded by the `ggcyto_GatingSet` and `ggcyto_flowSet` classes. The cytometry data structures are processed by the "`+`" operator, extracting and data and generating ggplot-compatible objects in the following sequence:

```
 1. Appropriate data structures are created from the arguments of t
he `geom_gate()` layer.
 2. The appropriate `geom_density` or `geom_point` layers are creat
ed from `geom_overlay()` to display the data.
 3. Cell population statistics are computed labels are created from
the `geom_stats()` layer.
 4. Marker or channel names are displayed on the axes and the corre
sponding label layers are created.
```

2.5 Principles of ggcyto.

In **ggplot** users map plot aesthetics to variables and build a plot in layers. **ggCyto** follows those conventions, but the application domain constrains requirements so many options are set to sensible defaults. A user specifies a *data source* (a flowSet or flowFrame of ungated data, or a GatingSet or GatingHierarchy of gated data), map plot axes to flow parameters (e.g. channels or markers), specify which cell population to plot, specify an axis transformation, and optionally add one or more gates to a plot. Many of these quantities are complex objects defined in the core cytometry data structures in BioConductor (Figure 1).

2.6 Lazy loading of data

For large data sets ggcyto implements context-dependent lazy loading. When plotting with the **ggcyto** or **autplot** APIs, data loading is automatically deferred to later stages of plotting.

2.7 Additional strategies for plotting large data

The latest version of **ggcyto** (1.9.4) provides speeds up plotting of large data sets through a subsampling strategy. Additional arguments to `autoplot` and `ggcyto` named `sample.ratio` and `sample.threshold` specify what proportion of events to subsample (default 1%, 0.01) and at what total number of events should subsampling kick in (default: 100,000 events). This substantially speeds up plotting of large cell populations such as lymphocytes (https://github.com/RGLab/ggcyto/issues/32).

2.8 Data sets

This document use the "graft vs. host disease" (`GvHD`) data set from the `flowCore` package on BioConductor (https://doi.org/doi:10.18129/B9.bioc.flowCore)), and part of the FlowCAP Lyoplate data that is in the `flowWorkspaceData` package, also on BioConductor (https://doi.org/doi:10.18129/B9.bioc.flowWorkspaceData)). Additional resources for cytometry data may be available via Bioconductor's **AnnotationHub** and **ExperimentHub** resources (Maintainer (2017), Morgan (2017))

3 Reproducing figures from the original manuscript.

The original manuscript shows three sets of plots created with ggcyto . To reproduce these, we load the GvHD data and use the code presented in the published figure to generate the plots.

```
#load the GvHD Data
data(GvHD)
#Use system.file to point to the location of the GatingSet in flowW
orkspaceData.
dataDir <- system.file("extdata",package="flowWorkspaceData")
#load_gs from the flowWorkspace package loads the gs_bcell_auto Gat
ingSet.
#list.files provides the full path to the GatingSet.
#The data loaded is the B cell autogated lyoplate data.
gs <- load_gs(list.files(dataDir, pattern = "gs_bcell_auto",full =
TRUE))

# Below we associate a transformation (FlowJo biexponential) with t
he GatingSet since it did
# not have one. It is applied to all channels except FS and SSC (fi
rst two columns)
gs@transformation <- transformerList(colnames(gs)[-(1:2)], flowJo_b
iexp_trans())

# For the GvHD data we select subjects 5 and 7, and Visit 5 and 6.
# We extract those sample "name"s and use that to subset the GvHD f
lowSet.
fs <- GvHD[subset(pData(GvHD), Patient %in%5:7 & Visit %in% c(5:6))
[["name"]]]

# We attach meaningful visit and subject identifiers to the samples
 via the phenoData.
pd <- pData(fs)
pd$Visit <- paste("visit",pd$Visit)
pd$Patient <- paste("patient",pd$Patient)
pData(fs) <- pd
```

3.1 Reproducing the plots from Figure 1.

3.1.1 The autoplot API

Here autoplot is used to visualize the populations named “CD3” and “CD19” from the GatingHierarchy . Since cell populations are defined by gates, the vector of population names is passed to the gate argument of autoplot when the data are a GatingSet (first argument). The bins argument specifies the size of the hexagonal binning grid.

The geom_overlay layer allows us to highlight an additional cell population on top of an existing plot. In this case, we are highlighting the “IgD+CD27+” population.

All cell populations in the gating hierarchy can be printed via the getNodes() API from flowWorkspace .

```
autoplot(gs[1], gate = c("CD3","CD19"), bins = 64) +
  geom_overlay(data = "IgD+CD27+",size=0.25)
```

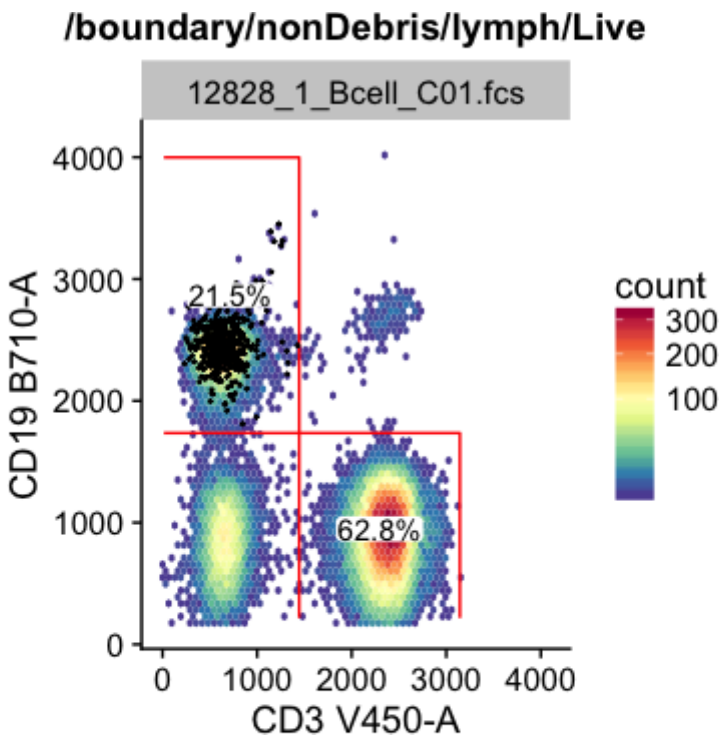

When using the autoplot API, most plotting decisions are set to sensible defaults, depending on the context of the input (e.g. whether the data is a flowFrame or flowSet, or GatingSet or GatingHierarchy). When the `autoplot` API takes a **flowFrame** or **flowSet**, the user specifies the **channels** or **markers** to plot on the axes, rather than a cell population, since these structures represent ungated data.

For GatingSets and GatingHierarchies, the first input is a **cell population name** (or vector of several populations, provided they are defined in the same dimensions, see below), stored in the data source. This determines the selection of axes for plotting, the subset of the cells that are plotted (generally the cells in the parent of the selected population are plotted), the data transformation (by default the transformation stored in the data source is used and raw data values are plotted on tick marks with a non-linear spacing, analogous to a logarithmic plot). Any gates that define the chosen cell populations are drawn. When using autoplot, the additional parameter `axis_inverse_trans` switches between the transformed and untransformed data scale. Finally, the proportion of the gated populations (as a fraction of the parent) are displayed on the plot.

The plots that are constructed are also “aware” of the dimensionality of a cell population. If a cell population is defined (e.g. gated) in only one dimension, **ggcyto** will produce a 1D density, whereas if it is defined in two dimensions, a 2D density estimate of the cells (using **geom_hex**) will be produced. Passing in a single flowFrame with no markers creates a set of 1D densities for each marker in the data (see below).

3.1.2 The `ggcyto` API.

The ggcyto API allows for more control and flexibility in producing plots.

```
# The mapping of channels to axes is explicit via the mapping = aes
(). argument.
# The subset argument specifies the cell subset / subset of the dat
a to plot.
# A geom_hex layer with the bins argument specifies the type of plo
t.
# A geom_gate layer specifies which gates / cell populations to add
 to plot.
#   the user must ensure they are defined in the same channels as t
he mapping.
# The geom_stats layer specifies here that we want to show the coun
ts.
#    The "percent" argument to "type" in "geom_stats" is also allow
ed.
ggcyto(gs[1:2], mapping = aes(x="IgD",y="CD27"), subset = c("CD19an
dCD20")) +
  geom_hex(bins=128) +
  geom_gate(c("IgD+CD27+","IgD+CD27-","IgD-CD27+","IgD-CD27-")) +
  geom_stats(type = "count")
```

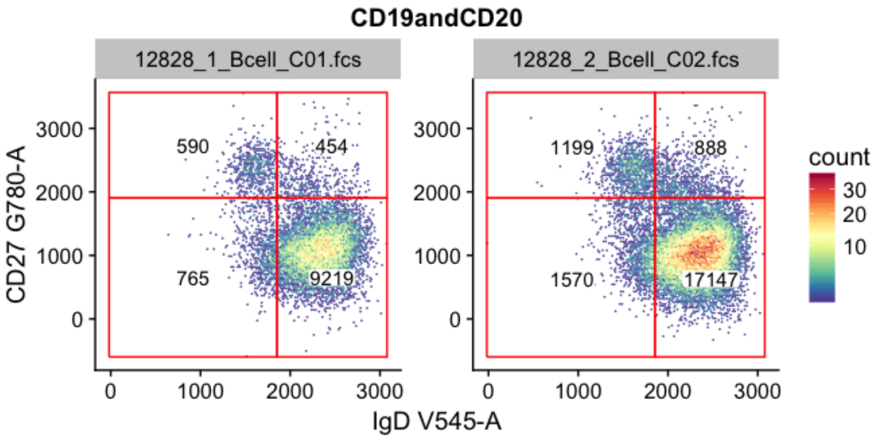

Above, the **geom_gate** layer will add one or two-dimensional gates to a plot and accepts a **gate** object (eg. “rectangleGate”) or a *filterList* (collection of gate objects) defined in the **flowCore** package, or **named cell population** if using a gated data source. The most recent version of ggcyto (1.9.4) also supports **quadGate**. Cells from a different cell population can be highlighted and overlaid on an existing plot using the **geom_overlay** (see below) layer to produce an effect of backgating (i.e. visualizing a defined cell population on data projected onto a different set of parameters). Cell population summaries such as cell counts, percentages, or proportions can be added to figures of gated data using **geom_stats**, which takes the name of a gated population in a `GatingSet` , or a `filterList` . With no input, ggcyto tries to parse the gate information from the first **geom_gate** layer.

Data transformations can be performed using the custom **scale_x** and **scale_y** layers. Common cytometry specific transformations are supported, including the default parameterization of the biexponential and the default parameterization of the hyperbolic arcsine transforms implemented in FlowJo, and the logicle and default hyperbolic arcsine transform parameterizations implemented in `flowCore` .

Another useful feature is the overloaded ‘%+%’ method, which is used to replace the data used in a (potentially complex) plot construct. For example, one may wish to produce a graphic using one GatingSet object, then the same graphic using a

different data from a separate GatingSet object. To produce the new plot, the ‘%+%’ method is applied with the old plot object on the left and the new data on the right hand side of the operator (see below).

3.1.3 Transforming the data.

The `scale_x_...` and `scale_y_...` layers allow users to alter the transfomration used for data visualization on the fly. Supported transformations are `..._flowCore_fasinh` , `..._flowJo_biexp` , `..._flowJo_fasinh` *, `..._logicle` .

```
#Use the first GvHD sample
fr <- GvHD[[1]]

# Autoplot is called on ungated data.
# In this case the x argument is a channel rather than "gate".
# The cowplot package is used to provide a theme.
p = autoplot(fr, x = "FL1-H") + theme_cowplot(font_size = 18)
print(p)
```

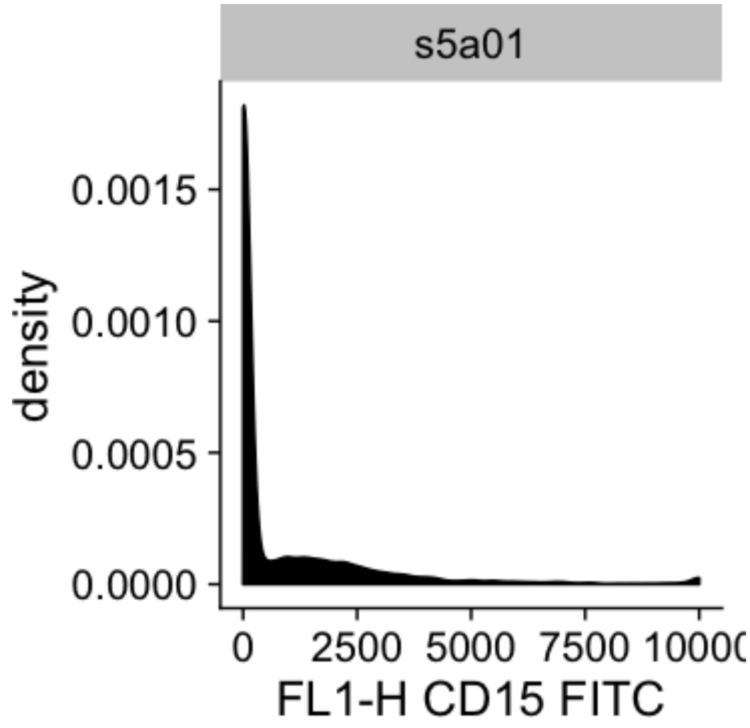


```
#add an inline transformation: flowCore logicle scale
p + scale_x_logicle()
```

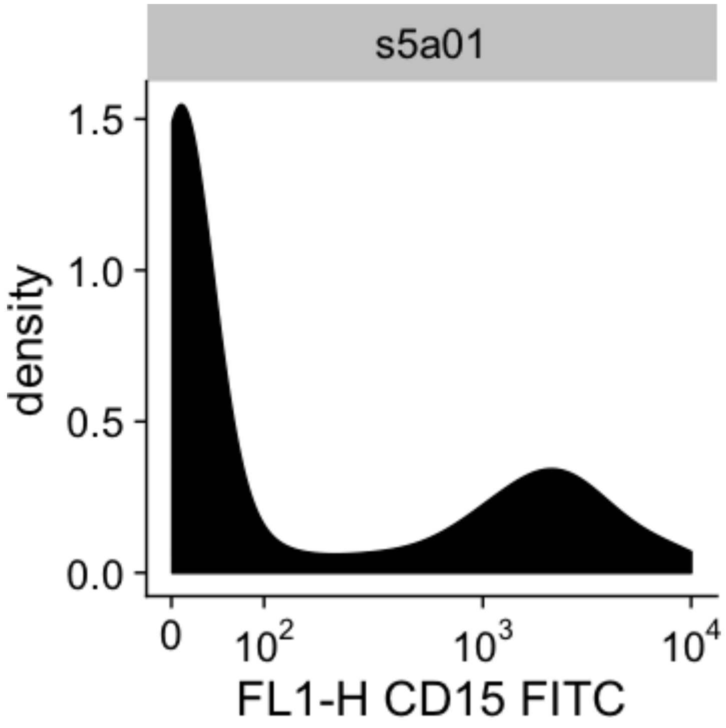


```
# add an inline transformation: flowJo fasinh
p + scale_x_flowJo_fasinh()
```

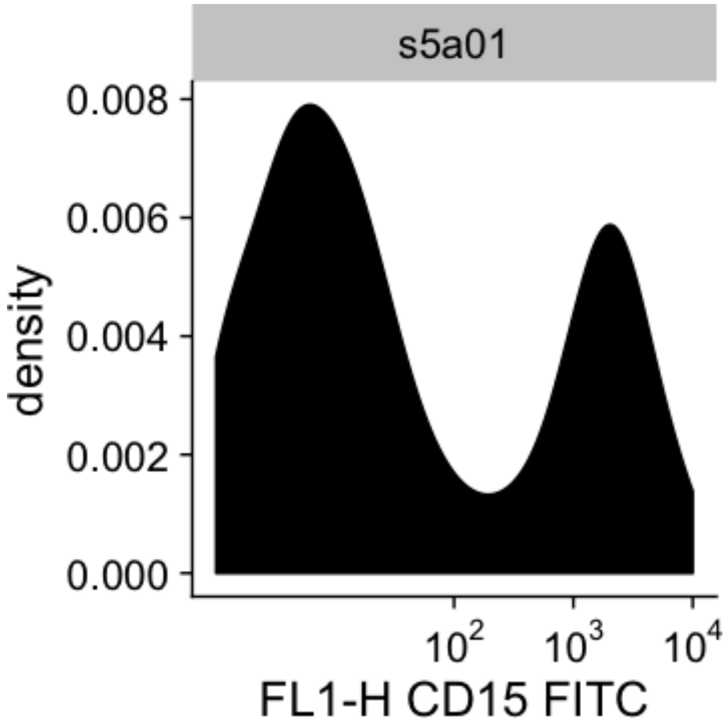

4 Additional ggcyto features.

The ggcyto package is built on `ggplot2` and uses the “grammar of graphics”. Below we demonstrate how its features can be used to easily generate high quality plots of cytometry data.

4.1 Context-aware plots.

Following common ggplot2 usage, ggcyto defines an autoplot() method. Calling autoplot() on a supported data structure automatically results in a default plot that is sensible for the data being displayed.

For example, supplying a flowSet and a channel name result in a 1D density plot using geom_density . If more than one sample is passed in, a faceted plot of all samples is created. The facet_grid and facet_wrap geoms can be used on variables in the pData slot of the flowFrame or flowSet object. Below we generate a faceted 1D density grid of the forward scatter channel from the first four samples of the GvHD data.

```
# we select the first four samples
# We plot the forward scatter channel.
# facet_grid is specified to facet by Patient and Visit, which are
 defined in
# the pData() slot of the flowSet.
autoplot(fs[1:4], x="FSC-H") + facet_grid(Patient~Visit)
```

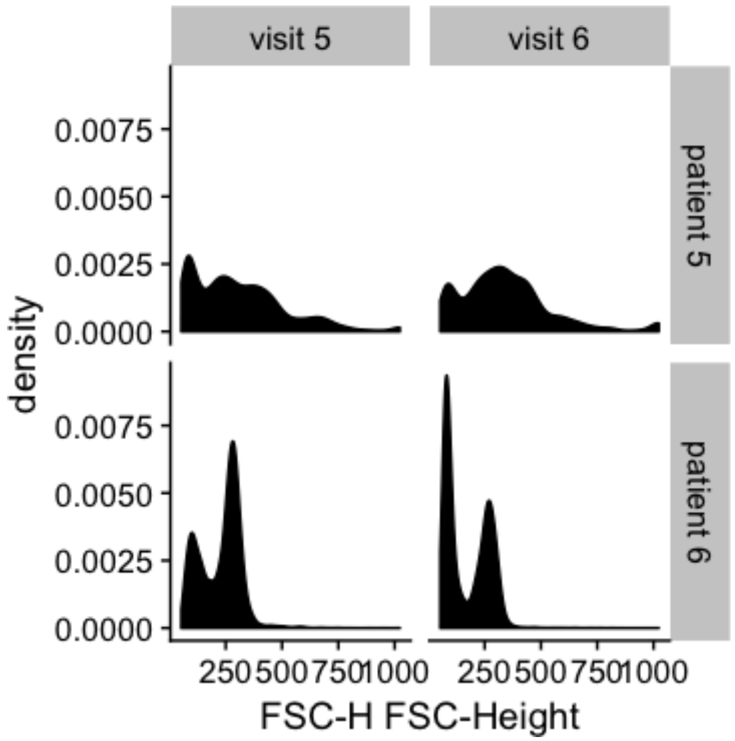

4.2 Changing geom s.

An existing plot can be easily modified to use a different geometry by appending the appropriate geom to the plot construction call, as below.

```
#geom_histogram replaces the default geom_density.
#again we are faceting by Patient and Visit.
autoplot(fs[1:4], x = "FSC-H") + facet_grid(Patient~Visit) + geom_h
istogram()
```

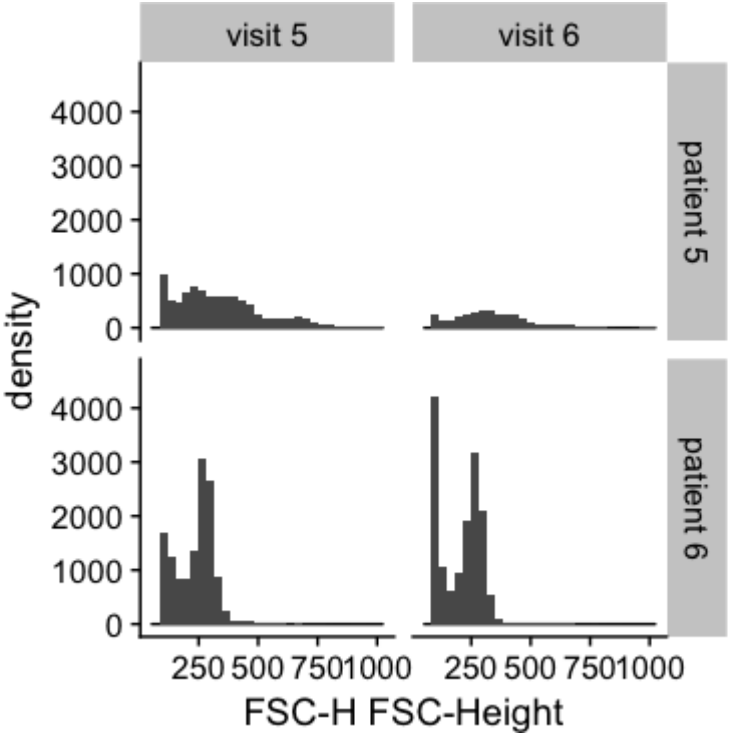

4.3 Using gated data.

Calling `autoplot` on a `GatingSet` or `GatingHierarchy` and supplying a **population name** (also an alias for a **gate** since the former are defined by the latter) results in a 1D or 2D density (depending on how the gate is defined) using `geom_hex` or `geom_density`. Provided the specified gates are compatible (defined on the same channels, have the same dimensionality), they will be projected onto the plot:

```
# CD9 and CD19 are gates / populations defined in the gating set.
autoplot(gs[[1]], gate = c("CD3", "CD19"), bins = 64)
```

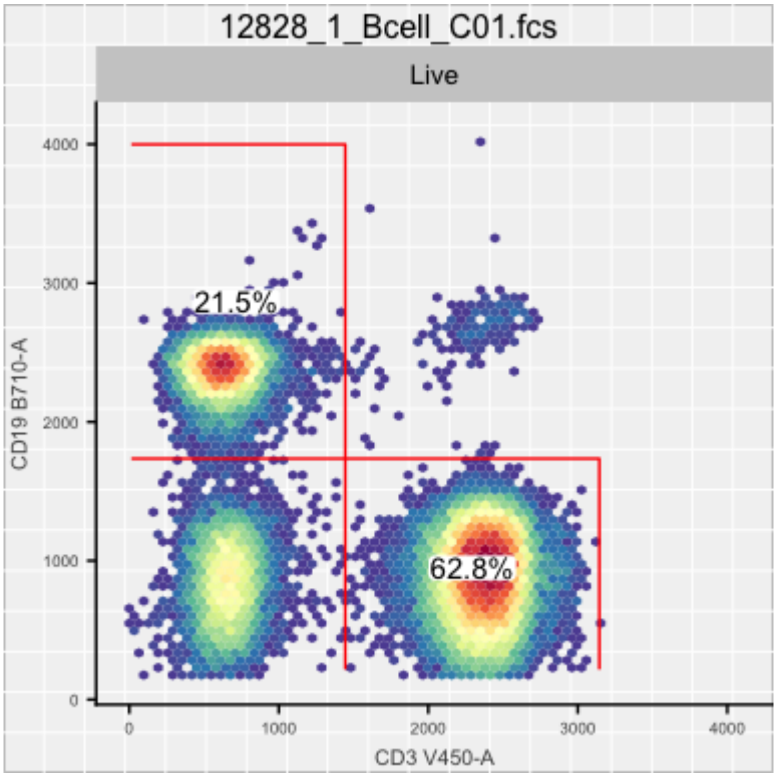

We can visualize the tree of cell populations using the `plot` method defined in `flowWorkspace`. `bool=TRUE` ensures boolean gates are also plotted.

```
# the tree shows that CD3 and CD19 (used above) are cell population
s.
plot(gs[[1]],bool=TRUE)
```

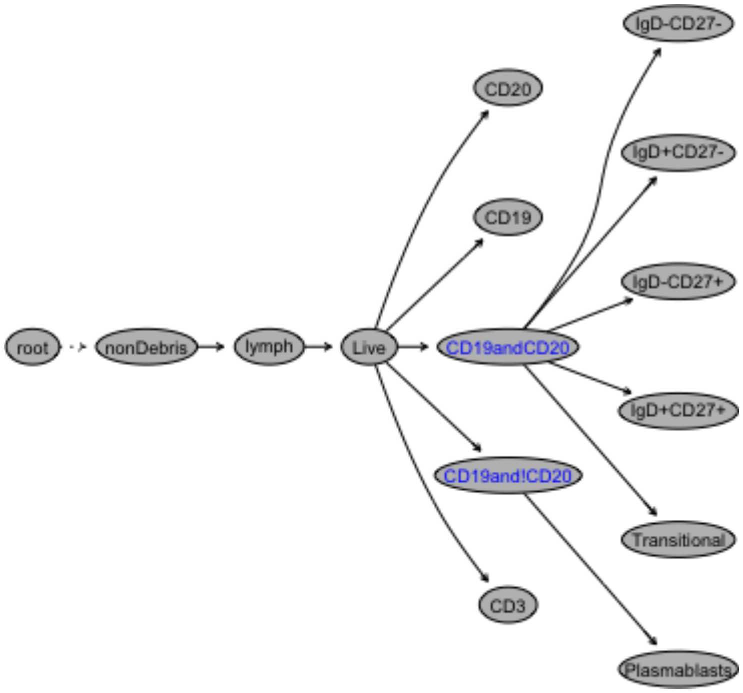

4.4 Visualizing back-gating.

Different populations can be overlaid on the same plot using `geom_overlay`, a ggcyto-specific `geom`. This is analogous to viewing back-gated cell populations. Below we highlight the IgD+CD127+ cells on the CD19 and CD3 projection of a

sample from the Lyoplate B cell panel.

```
# we add an overlay of IgD+CD27+ cells on top of the display of CD3
 and CD19 cells.
# We see they are CD19 positive
autoplot(gs[1], gate = c("CD3","CD19"), bins = 64) +
  geom_overlay("IgD+CD27+",size=0.5)
```

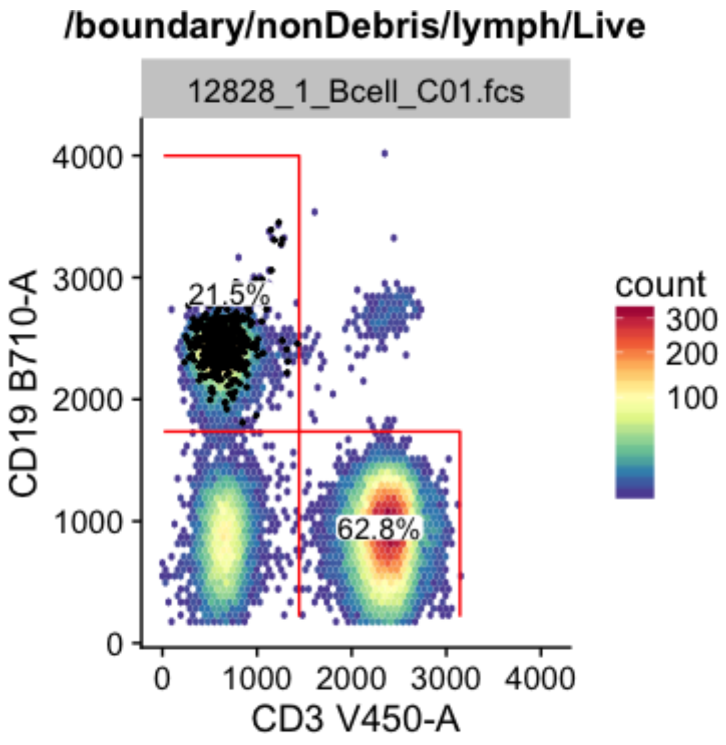

4.5 Transforming the axes.

The x and y axis scales are transformed above but show the raw data values. These can be changed to show the transformed data values on a linear scale using the `axis_inverse_trans` argument to `autoplot`. Below, we see the data are in 4096 “channel” space, commonly used by FlowJo and other tools.

```
# The axis_inverse_trans argument allows us to display the x and y
 axes on the scale
# which is used in the GatingSet. In this case, the data are in 409
6 "channel" space.
# When used with tools like openCyto (which acts on the data in the
 GatingSet), this is useful for
# passing gate ranges and other parameters to gating algorithms.
autoplot(gs[1], gate = c("CD3","CD19"), bins = 64, axis_inverse_tra
ns = FALSE) +
  geom_overlay("IgD+CD27+",size=0.5)
```

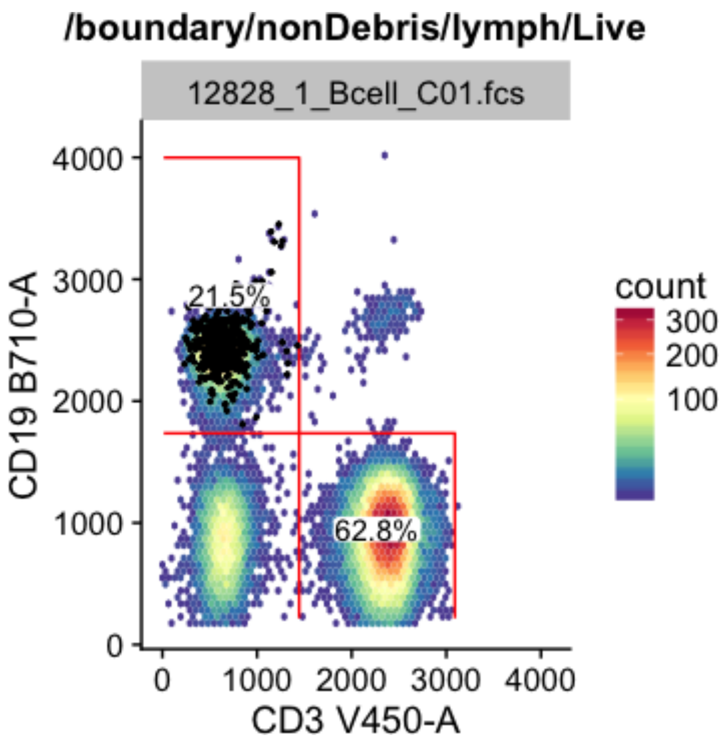

4.6 Using the `ggcyto` interface.

The same plots created with `autoplot` can be generated using `ggcyto()`. Below, we show how to create the previous plot using the `ggcyto` API. Note below, the gate names and channel names are the same, but this is not necessarily always the case.

```
# mapping specifies the channels to plot on the x and y axes.
# geom_gate specifies the gate names / population name
# (note they just happen to be the same as the channel names but th
is is
# not necessarily always the case).
# geom_stats specifies that we want to plot the "percent" for each
 cell population.
# The x and y axis_inverse_trans reverse the axis transformation an
d plots the data in "channel" space (in this case).
ggcyto(gs[1], mapping = aes(x="CD3",y="CD19"),subset= "Live") +
  geom_hex(bins=64) +
  geom_gate(c("CD19","CD3")) +
  geom_stats(type = "percent") +
  axis_x_inverse_trans() +
  axis_y_inverse_trans()
```

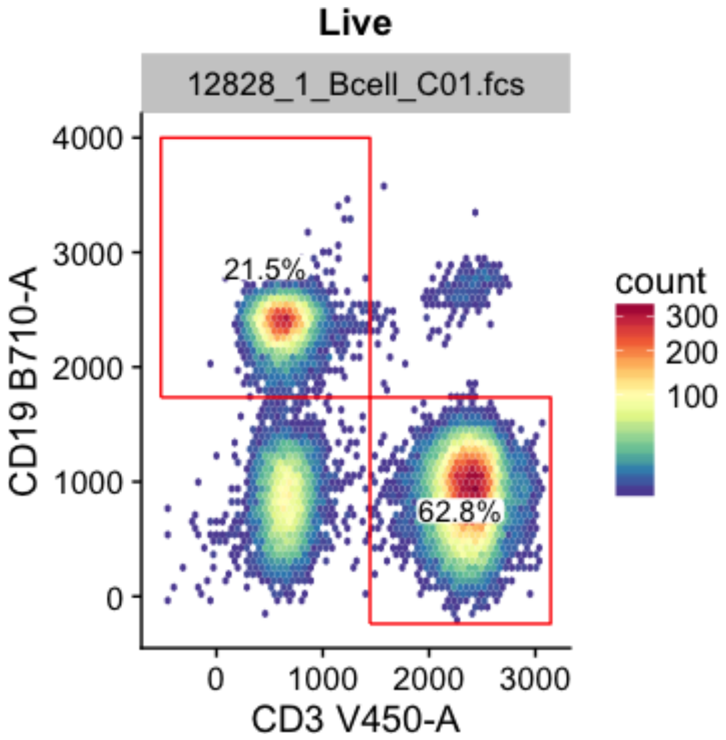

4.7 Faceting plots by experimental metadata.

We load the lyoplate data for the T cell panel. the data are available on ImmuneSpace (http://www.immunespace.org) from this link (https://immunespace.org/_webdav/HIPC/Lyoplate/%40files//gated_data/pop_renamed/manual-gslist-tcell.tar.gz) (free ImuneSpace sign up and login required) (Sauteraud et al. (2016), Brusic et al. (2014)).

```
# load the Lyoplate gatingSet. Here it's been downloaded and stored
 locally. The user reproducing this workflow should update their li
nk.
gs2 <- rbind2(load_gslist("~/Dropbox/GoTeam/Members/Phu/manuscript
s/GGCyto/Paper version 1/gslist-tcell/"))

# A transformation is added to the GatingSet so that the axis_inver
se_trans and other arguments work as expected.
gs2@transformation = transformerList(colnames(gs2)[-(1:2)], flowJo_
biexp_trans())
```

The Lyoplate dataset for the T cell panel contains three biological samples. The samples were distributed across seven centers, an each center ran three technical replicates of each sample, as indicated in the metadata.

name	Center	Sample	Replicate
12828_1_Tcell_A01.fcs	NHLBI	12828	1
12828_2_Tcell_A02.fcs	NHLBI	12828	2
12828_3_Tcell_A03.fcs	NHLBI	12828	3
1349_1_Tcell_A04.fcs	NHLBI	1349	1
1349_2_Tcell_A05.fcs	NHLBI	1349	2

name	Center	Sample	Replicate
1349_3_Tcell_A06.fcs	NHLBI	1349	3
1369_1_Tcell_A07.fcs	NHLBI	1369	1
1369_2_Tcell_A08.fcs	NHLBI	1369	2
1369_3_Tcell_A09.fcs	NHLBI	1369	3
12828_1_A1_A01.fcs	Yale	12828	1
12828_2_A2_A02.fcs	Yale	12828	2
12828_3_A3_A03.fcs	Yale	12828	3
1349_1_A4_A04.fcs	Yale	1349	1
1349_2_A5_A05.fcs	Yale	1349	2
1349_3_A6_A06.fcs	Yale	1349	3
1369_1_A7_A07.fcs	Yale	1369	1
1369_2_A8_A08.fcs	Yale	1369	2
1369_3_A9_A09.fcs	Yale	1369	3
TCELL 22013_12828_001.fcs	UCLA	12828	1
TCELL 22013_12828_002.fcs	UCLA	12828	2
TCELL 22013_12828_003.fcs	UCLA	12828	3
TCELL 22013_1349_001.fcs	UCLA	1349	1
TCELL 22013_1349_002.fcs	UCLA	1349	2
TCELL 22013_1349_003.fcs	UCLA	1349	3
TCELL 22013_1369_001.fcs	UCLA	1369	1
TCELL 22013_1369_002.fcs	UCLA	1369	2
TCELL 22013_1369_003.fcs	UCLA	1369	3
T_CELL_12828_001_P1.fcs	CIMR	12828	2
T_CELL_12828_002_P1.fcs	CIMR	12828	3
T_CELL_12828_P1.fcs	CIMR	12828	1
T_CELL_1349_001_P1.fcs	CIMR	1349	2
T_CELL_1349_002_P1.fcs	CIMR	1349	3
T_CELL_1349_P1.fcs	CIMR	1349	1
T_CELL_1369_001_P1.fcs	CIMR	1369	2
T_CELL_1369_002_P1.fcs	CIMR	1369	3
T_CELL_1369_P1.fcs	CIMR	1369	1

name	Center	Sample	Replicate
lot 12828_A1_A01.fcs	Miami	12828	1
lot 12828_A2_A02.fcs	Miami	12828	2
lot 12828_A3_A03.fcs	Miami	12828	3
lot 1349_A4_A04.fcs	Miami	1349	1
lot 1349_A5_A05.fcs	Miami	1349	2
lot 1349_A6_A06.fcs	Miami	1349	3
lot 1369_A7_A07.fcs	Miami	1369	1
lot 1369_A8_A08.fcs	Miami	1369	2
lot 1369_A9_A09.fcs	Miami	1369	3
12828_1_T CELL.fcs	Baylor	12828	1
12828_2_T CELL.fcs	Baylor	12828	2
12828_3_T CELL.fcs	Baylor	12828	3
1349_1_T CELL.fcs	Baylor	1349	1
1349_2_T CELL.fcs	Baylor	1349	2
1349_3_T CELL.fcs	Baylor	1349	3
1369_1_T CELL.fcs	Baylor	1369	1
1369_2_T CELL.fcs	Baylor	1369	2
1369_3_T CELL.fcs	Baylor	1369	3
1228-1_A1_A01.fcs	Stanford	12828	1
1228-2_A2_A02.fcs	Stanford	12828	2
1228-3_A3_A03.fcs	Stanford	12828	3
1349-1_A4_A04.fcs	Stanford	1349	1
1349-2_A5_A05.fcs	Stanford	1349	2
1349-3_A6_A06.fcs	Stanford	1349	3
1369-1_A7_A07.fcs	Stanford	1369	1
1369-2_A8_A08.fcs	Stanford	1369	2
1369-3_A9_A09.fcs	Stanford	1369	3

The data was gated to identify CD4 and CD8 memory T cells:

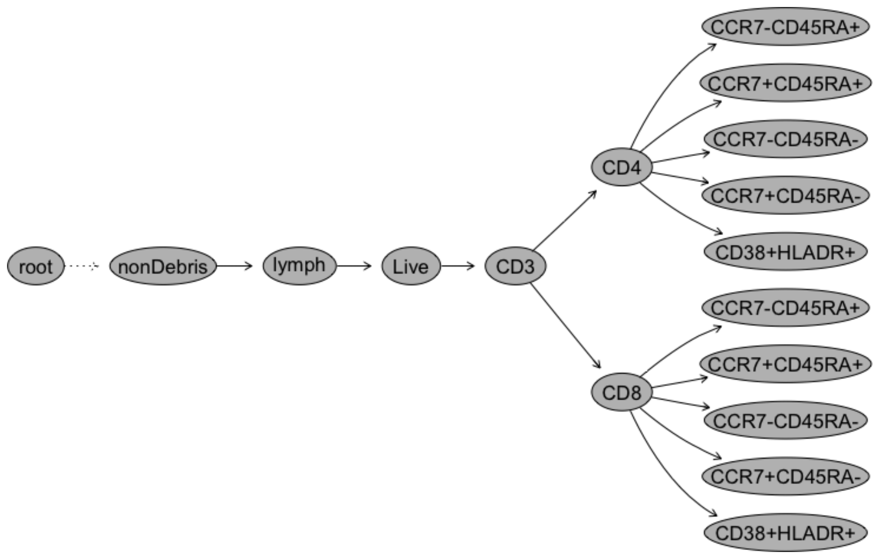

We can visualize variation across replicates and centers for sample #1349. The plot is built up using the `ggcyto()` API. Note we use `axis_x_inverse_trans` and `axis_y_inverse_trans` to transform the axes tick marks to a non-linear scale and display the raw data values.

The plot shows clearly that center-to-center variability is greater than variability across technical replicates.

```
# subset the relevant sample
p = ggcyto(subset(gs2, Sample=="1349")
# map CCR7 to x and CD45RA to y
        ,aes(x = CCR7,y=CD45RA)
# plot the CD4 subset of cells
        ,subset="CD4") +
# display the data as a hexagonally binned density with 64 bins.
        geom_hex(bins = 64) +
# plot the CCR7+/CD45RA- gate. Note we specify the parent as CD4+ s
ince the gate also exists for CD8+
        geom_gate("CD4/CCR7+CD45RA-") +
# We want to show population statistics.
        geom_stats() +
# We facet by replicate and center.
        facet_grid(Replicate ~ Center) +
# add a title
        labs(title="sample 1349 CD4 central memory T cells, by cent
er and replicate") +
# options to set the axis limits to the "instrument" range. Could a
lso be "data".
        ggcyto_par_set(limits = "instrument") +
# invert the axis transformation to show raw data on x and y
        axis_x_inverse_trans() +
        axis_y_inverse_trans()
p
```

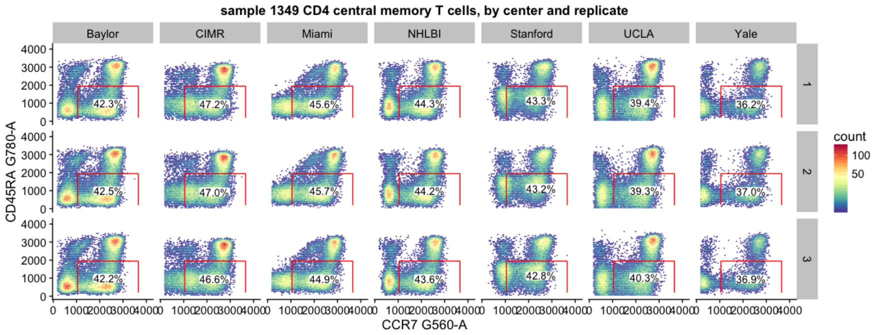

4.8 Substituting data with the %+% method.

Here we use the %+% method to substitute the data in the plot. We'll plot only a subset of the centers.. specifically Baylor and CIMR.

```
# We use the %+% method to "substitute" a different set of data wit
h the same plot parameters.
# The right hand side of %+% takes a GatingSet object in this case,
 but could be a flowSet, flowFrame, or GatingHierarchy, depending o
n the nature of "p".
p %+% subset(gs2,Center%in%c("Baylor","CIMR"))
```

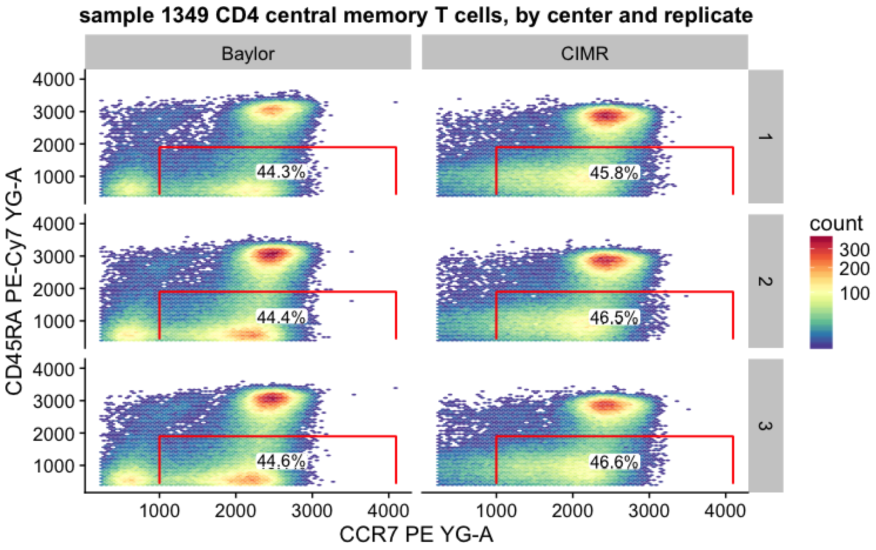

4.9 Faceting all markers.

As mentioned in the overview, when we pass in a single flowFrame to `autoplot` with no markers specified, we obtain a series of faceted 1D densities, one for each marker in the sample.

```
autoplot(fs[[1]])
```

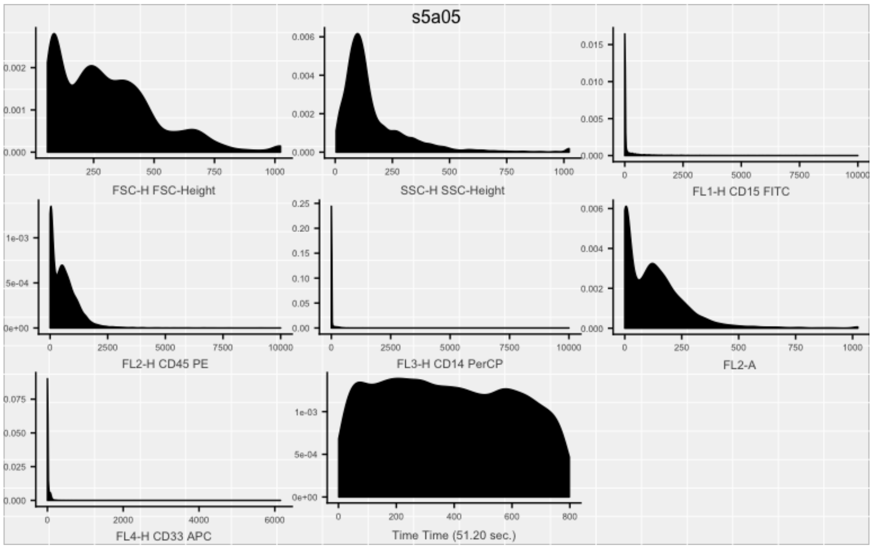

4.10 Future Work

Since ggcyto utilizes ggplot's `fortify()` method under the hood, users must take care in plotting large cell populations that result in manipulating large tables of data. This could be resolved by a C++ back-end for processing the data and by leveraging parallelization, such as that implemented in the cytolib package (https://github.com/RGLab/cytolib). For now, the latest version of ggcyto allows subsampling (https://github.com/RGLab/ggcyto/issues/32) of the data as described earlier in this document.

References

Brusic, Vladimir, Raphael Gottardo, Steven H Kleinstein, Mark M Davis, and HIPC steering committee. 2014. "Computational Resources for High-Dimensional Immune Analysis from the Human Immunology Project Consortium." *Nat. Biotechnol.* 32 (2): 146–48.

Finak, Greg, Wenxin Jiang, Kevin Krouse, Chungwen Wei, Ignacio Sanz, Deborah Phippard, Adam Asare, Stephen C De Rosa, Steve Self, and Raphael Gottardo. 2014. "High-Throughput Flow Cytometry Data Normalization for Clinical Trials." *Cytometry A* 85 (3). Wiley Online Library: 277–86.

Hahne, Florian, Nolwenn LeMeur, Ryan R Brinkman, Byron Ellis, Perry Haaland, Deepayan Sarkar, Josef Spidlen, Errol Strain, and Robert Gentleman. 2009. "FlowCore: A Bioconductor Package for High Throughput Flow Cytometry." *BMC Bioinformatics* 10 (1). BioMed Central Ltd: 106.

Jiang, Mike, Greg Finak, N Gopalakrishnan, Maintainer M Jiang, Imports Biobase, and Collate Allgeneric R Allclasses R AllFunctions. 2011. "Package 'ncdfFlow'."

Maintainer, Bioconductor Package. 2017. *ExperimentHub: Client to Access Experimenthub Resources*.

Morgan, Martin. 2017. *AnnotationHub: Client to Access Annotationhub Resources*.

Sauteraud, Renan, Lev Dashevskiy, Greg Finak, and Raphael Gottardo. 2016. "ImmuneSpace: Enabling Integrative Modeling of Human Immunological Data." *The Journal of Immunology* 196 (1 Supplement): 124.65–124.65.

Wickham, H. 2009. *Ggplot2: Elegant Graphics for Data Analysis*. Use R! Springer New York.
